# Absence of *JAK2* V617F-mutated polycythemia vera in obstructive sleep apnea-associated erythrocytosis

**DOI:** 10.1016/j.sleepx.2022.100058

**Published:** 2022-10-15

**Authors:** Stephen E. Langabeer

**Affiliations:** Cancer Molecular Diagnostics, St.James's Hospital, Dublin, Ireland

**Keywords:** Obstructive sleep apnea, Erythrocytosis, Polycythemia vera, Molecular diagnostics

Dear Editor

The prevalence of obstructive sleep apnea (OSA) is conservatively estimated at 9% in women and 17% among men aged 50–70 years [1]. The resultant intermittent hypoxia results in a widely recognised secondary erythrocytosis [2]. In contrast, the most frequent form of primary erythrocytosis is the myeloproliferative neoplasm of polycythemia vera (PV) that is characterised by the presence of the *JAK2* V617F somatic mutation in approximately 98% of cases [3]. Leucocytosis, thrombocytosis, splenomegaly, bone marrow erythroid hyperplasia and a low serum erythropoietin are also commonly present in PV patients. Despite the above distinction, OSA patients with an erythrocytosis are sporadically referred for molecular detection of the *JAK2* V617F, presumably in order to exclude co-existing PV. An audit was therefore performed to determine the clinical and laboratory impact of *JAK2* V617F testing in patients with OSA-associated erythrocytosis.

At a center for hematological malignancy molecular diagnostics that receives greater than 2000 *JAK2* V617F diagnostic tests per annum, from January 2006 to June 2022 inclusive, 73 requests for *JAK2* V617F mutation status were identified with clinical details provided of OSA and either erythrocytosis and/or raised hemoglobin and/or raised hematocrit. The median age of OSA patients was 67 years (range 32–90 years) of which 37 patients (50.6%) were male. The *JAK2* V617F mutation was detected by an allele-specific PCR technique unchanged throughout the audit period. The *JAK2* V617F mutation was not detected in any of the 73 cases analysed.

While OSA is a prevalent cause of secondary erythrocytosis [4] the possibility exists of OSA-associated erythrocytosis and concomitant PV: a single case is identified in the literature with the PV unconfirmed by absence of *JAK2* mutation analysis [5]. Despite molecular investigation for the *JAK2* V617F in OSA-associated erythrocytosis not affecting laboratory workload, this audit indicates that routine testing for PV is unnecessary.

## Ethical considerations

This non-interventional, retrospective study was performed as routine standard of care and in accordance with the World Medical Association Declaration of Helsinki. Patient consent was required at the referring center.

## Declaration of competing interest

The authors declare that they have no known competing financial interests or personal relationships that could have appeared to influence the work reported in this paper.
